# EPIC: Event Prototyping via Information Constrained graph learning for personalized cancer driver gene prediction

**DOI:** 10.1093/bioinformatics/btag229

**Published:** 2026-07-07

**Authors:** Sang-Pil Cho, Young-Rae Cho

**Affiliations:** Department of Software, Yonsei University—Mirae Campus, Wonju-si, Gangwon-do, 26493, Republic of Korea; Department of Software, Yonsei University—Mirae Campus, Wonju-si, Gangwon-do, 26493, Republic of Korea; Department of Digital Healthcare, Yonsei University—Mirae Campus, Wonju-si, Gangwon-do, 26493, Republic of Korea

## Abstract

**Motivation:**

Precision oncology relies on accurately distinguishing patient-specific driver mutations from the vast background of passenger alterations. While graph-based computational methods have emerged as powerful tools for this task, they often struggle to preserve the distinct genomic context of individual mutations within complex biological networks. Consequently, subtle patient-specific driver signals are frequently obscured by dominant topological patterns, critically impeding the identification of individualized oncogenic events essential for personalized cancer therapy.

**Results:**

To address this, we propose EPIC, a novel framework for Event Prototyping via Information Constrained Graph Learning. Unlike traditional node-centric approaches, EPIC redefines driver prediction as a metric learning task in an event embedding space. We introduce an information-constrained learning strategy that imposes explicit geometric constraints on feature variance, effectively preventing feature collapse and ensuring that low-frequency driver signals are distinctively preserved. Experiments on large-scale cancer cohorts demonstrate that EPIC significantly outperforms established baselines. Notably, the model prioritizes low-frequency driver variants typically overlooked by population-based methods, mapping them to critical oncogenic mechanisms associated with drug resistance and metastasis. Furthermore, clinical actionability analysis confirms that EPIC substantially expands the patient population eligible for targeted therapies. EPIC provides a robust and context-aware solution for personalized cancer driver discovery, bridging the gap between genomic data and actionable therapeutic insights.

**Availability and implementation:**

The source code and datasets are available at https://github.com/spcho-dev/EPIC.

## 1 Introduction

Cancer is essentially a genetic disease driven by the accumulation of somatic mutations, yet only a small fraction of these—known as “drivers”—confer a selective growth advantage, while the majority remain neutral “passengers” (Vogelstein et al. 2013). Distinguishing these drivers is fundamental to precision medicine. While cohort-level methods (Raphael et al. 2014) have successfully identified recurrent drivers based on mutation frequencies, they inherently struggle with the high heterogeneity of cancer (Vogelstein et al. 2013). Since individual patients often harbor unique combinations of genomic alterations, these population-based approaches frequently fail to detect rare or patient-specific driver genes critical for personalized treatment (Pham et al. 2021).

To address this limitation, various personalized approaches have emerged, evolving from biological network analysis to sophisticated representation learning. Early network-based methods, such as DawnRank (Hou and Ma 2014) and PRODIGY (Dinstag and Shamir 2020), prioritized drivers by analyzing single-sample networks or dysregulated pathways. With the advancement of machine learning, IMCDriver (Zhang et al. 2021) and PersonaDrive (Erten et al. 2022) used inductive matrix completion and bipartite graphs to uncover latent gene–patient associations. More recently, to capture higher-order correlations, PCoDG (Zhang et al. 2024b) and PDRWH (Zhang et al. 2024a) employed hypergraph structures. However, despite these advances, significant challenges remain. Most approaches rely on static topological features or heuristic metrics that fail to fully capture the non-linear context of tumor biology. More critically, as these methods propagate information across complex networks, they often encounter a phenomenon where subtle, patient-specific driver signals are obscured by the dominance of common passenger mutations. This loss of distinct genomic context, frequently intensified by the over-smoothing effects in deep learning models (Li et al. 2018), severely limits the ability to identify rare but functionally relevant drivers in individual patients.

To overcome these challenges, we propose EPIC, a novel framework for Event Prototyping via Information Constrained Graph Learning. Unlike traditional node-centric approaches that operate on static features, EPIC redefines the problem as an “Event Prototyping” task. We conceptualize the occurrence of a mutation in a patient as a unique “event” characterized by the specific context of the patient’s gene expression profile. By constructing a heterogeneous graph, EPIC employs a Prototypical Metric Learning mechanism. Instead of simple binary classification, our model learns to map these events into an embedding space where their distances to learnable “Driver” and “Passenger” prototypes determine their priority scores. This approach allows for a more nuanced ranking of potential drivers based on their proximity to ideal oncogenic features.

Furthermore, to explicitly prevent patient-specific signals from being overwhelmed by noisy background data, we introduce an Information-Constrained learning strategy. By incorporating an “Information Flow” mechanism, EPIC imposes geometric constraints on the variance and diversity of feature representations. This ensures that the model preserves the distinct genomic context of each patient-gene event, protecting rare driver signals from feature collapse. We applied EPIC to five cancer datasets from The Cancer Genome Atlas (TCGA) (Weinstein et al. 2013) and demonstrated that our method consistently outperforms existing approaches. Crucially, our extensive analysis reveals that EPIC prioritizes low-frequency driver variants associated with critical oncogenic mechanisms and substantially expands the clinically actionable patient population. EPIC provides a robust and context-aware solution for personalized cancer driver discovery, offering new insights into the complex landscape of tumor biology.

## 2 Materials and methods

### 2.1 Data resources and preprocessing

In this study, we collected multi-omics data consisting of somatic mutation profiles and gene expression data from TCGA via the UCSC Xena platform (Goldman et al. 2020). Our study focuses on five major cancer cohorts: breast invasive carcinoma (BRCA), colon adenocarcinoma (COAD), head and neck squamous cell carcinoma (HNSC), lung adenocarcinoma (LUAD), and prostate adenocarcinoma (PRAD).

For each cohort, we used paired genomic data comprising somatic mutations and gene expression levels. The somatic mutation profiles consist of unified gene-level non-silent mutation calls, encoded as binary indicators where a value of 1 denotes the presence of a non-synonymous point mutation or indel, and 0 represents the wild type. The corresponding gene expression profiles were obtained from RNA-seq data quantified as RSEM normalized counts. These values were ${\;\text{log}\;}_{2}(x+1)$ transformed to reduce distributional skewness and ensure numerical stability during model training.

To ensure data consistency across modalities, we performed a rigorous preprocessing step. We intersected the samples and genes across the mutation and expression datasets, retaining only those entities present in both. Consequently, the final preprocessed dataset comprised a unified set of 18 616 genes across all cancer types. After filtering for samples with available paired data, the final cohort sizes were 789 samples for BRCA, 267 for COAD, 499 for HNSC, 509 for LUAD, and 494 for PRAD.

To capture biological interactions, we incorporated a global Protein–Protein Interaction (PPI) network from the STRING database (Szklarczyk et al. 2025). To ensure the reliability of the topological features used in our graph learning framework, we applied a stringent filtering criterion, retaining only high-confidence interactions with a combined score ≥0.85. Furthermore, we removed self-loops and duplicated edges to prevent information redundancy. The resulting refined PPI network consists of 9993 nodes and 242 176 edges.

### 2.2 Heterogeneous graph construction

**Figure 1:**
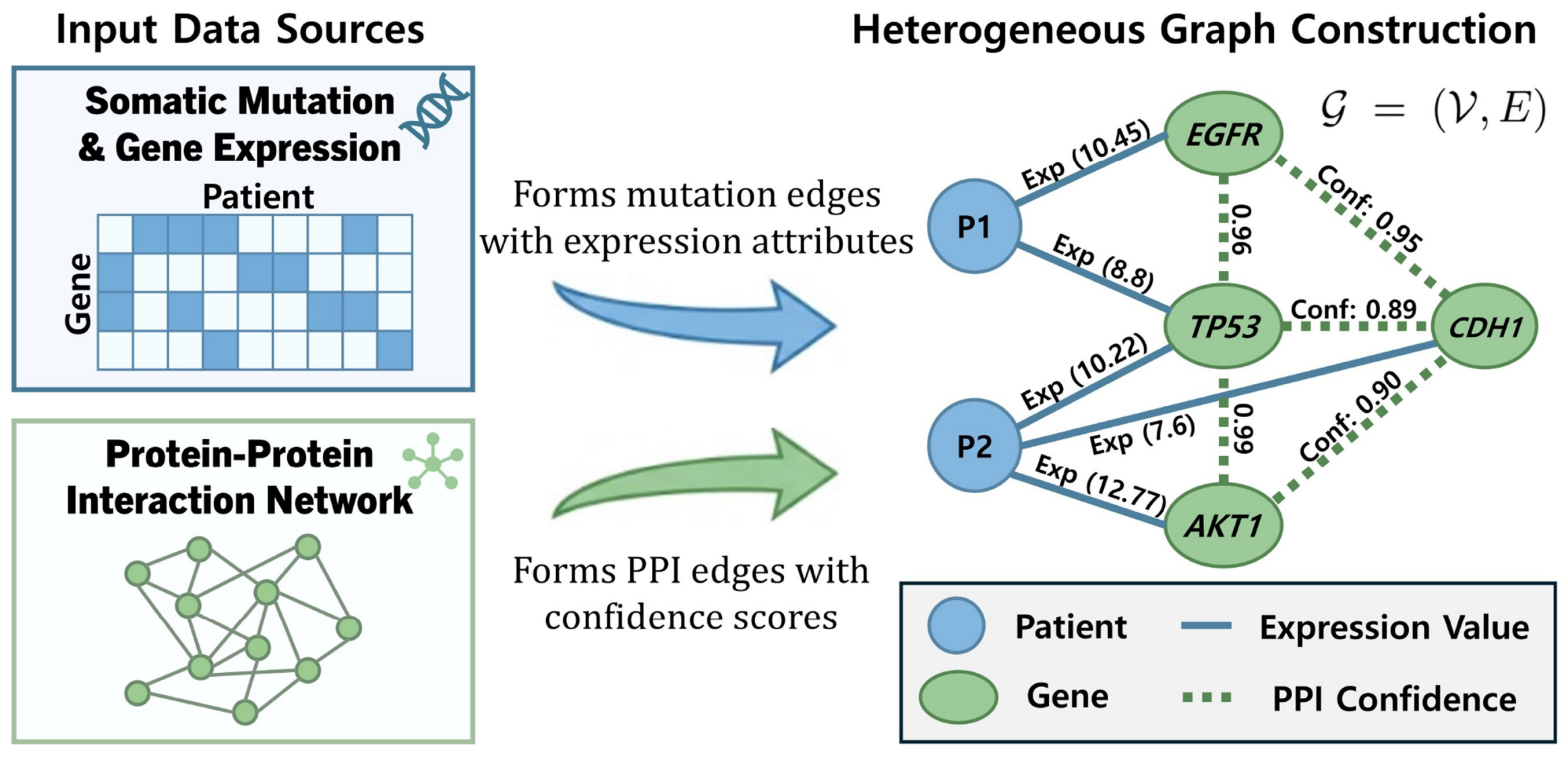
Schematic overview of the heterogeneous graph construction. The graph integrates patient nodes ($V_{p}$) and gene nodes ($V_{g}$). It features two edge types: patient-gene mutation edges weighted by gene expression, and gene–gene edges weighted by PPI confidence scores.

To model the complex biological interactions between patients and genes, we constructed a heterogeneous graph G=(V,E), as illustrated in Fig. 1. The node set V consists of two types of entities: patient nodes $u\in V_{p}$ and gene nodes $v\in V_{g}$. The edge set E integrates multi-omics data through two distinct relation types:

- **Gene–gene interactions (** $E_{\mathrm{PPI}}$ ): Derived from the filtered STRING database, these edges represent biological connections between proteins. To incorporate topological reliability, we utilize the interaction confidence scores as edge weights $w_{vv^{\prime }}^{\mathrm{PPI}}$.
- **Patient–gene associations (** $E_{\mathrm{Mut}}$ ): These edges are established when a somatic mutation is observed in gene *v* for patient *u*. Crucially, to integrate transcriptomic context, we assign the gene expression value as the edge attribute $x_{uv}^{\;\text{Exp}\;}$. This allows the model to differentiate between expressed and non-expressed mutations. Genes lacking high-confidence PPIs remain fully integrated into the learning process via these patient-gene mutation edges, ensuring their unique mutational contexts are actively processed during message passing.

All edge attributes and the initial node embeddings are projected into the hidden dimension *d* via linear encoders before being processed by the Graph Neural Network (GNN).

### 2.3 Information-constrained graph encoder

We used a GNN to learn low-dimensional representations of nodes. Specifically, we adopted the Graph Attention Network v2 (GATv2) (Brody et al. 2022) operator within a heterogeneous message-passing framework. The update rule for a node representation involves adding a residual term to the previous state. We formally define this residual update as the information flow vector $f_{i}^{\left(l\right)}$:


(1) $$f_{i}^{\left(l\right)}=\text{Dropout}\left(\text{ReLU}\left(\sum_{j\in N(i)}\alpha_{ij}W_{r}h_{j}^{\left(l\right)}\right)\right)$$

where $\alpha_{ij}$ denotes the attention coefficient computed using the encoded edge attributes. Consequently, the node update is given by $h_{i}^{\left(l+1\right)}=h_{i}^{\left(l\right)}+f_{i}^{\left(l\right)}$.

In personalized driver gene prediction, a critical challenge lies in distinguishing rare, patient-specific driver signals from the extensive background of prevalent passenger mutations. Standard deep GNNs, however, often suffer from over-smoothing, where node representations become homogenized, causing subtle but biologically significant driver events to be washed out by dominant topological trends. To prevent this loss of biological specificity and preserve the unique genomic context of each patient, we introduce an Information-Constrained learning strategy. Instead of allowing the information flow to degrade into noise or uniform patterns, we impose explicit geometric constraints on the collection of flow vectors f across all layers.

First, to promote stability and prevent exploding information updates, we regularize the variance of the flow norms via the variance loss:


(2) $$L_{\mathrm{var}}=\text{Var}\left(∥f_{i}^{\left(l\right)}{∥}_{2}\right)$$

This constraint encourages a consistent magnitude of information updates across nodes, mitigating the risk of noise amplification.

Second, to prevent representations from converging to a single direction (the mean field effect), we minimize directional collapse via diversity loss. We compute the mean flow direction ${\bar{u}}^{\left(l\right)}$ and penalize the cosine similarity of individual flows to this mean:


(3) $$L_{\mathrm{div}}=\frac{1}{N}\sum_{i=1}^{N}{\left(\text{cos}(f_{i}^{\left(l\right)},{\bar{u}}^{\left(l\right)})\right)}^{2}$$

By minimizing $L_{\mathrm{div}}$, EPIC forces the information flows to be orthogonal to the dominant trend, thereby preserving the unique, patient-specific context of each mutation event.

### 2.4 Event prototyping for driver prediction

Unlike traditional node-centric approaches classifying genes directly, EPIC formulates driver prediction as a metric learning problem within an “event” embedding space. We define a mutation “event” $z_{uv}\in R^{d}$ by fusing the learned patient embedding $h_{u}\in R^{d}$ and gene embedding $h_{v}\in R^{d}$ through a non-linear Multi-Layer Perceptron:


(4) $$z_{uv}=\text{MLP}(h_{u}⊕h_{v})$$

To evaluate the oncogenic potential of these events, we introduce two learnable prototype vectors: $p_{\mathrm{driver}}\in R^{d}$, representing the ideal features of a driver mutation, and $p_{\mathrm{passenger}}\in R^{d}$, representing those of a neutral passenger mutation. These prototypes are randomly initialized and jointly optimized with the model parameters via backpropagation to serve as the ideal anchor points for each class in the latent space. The priority score $S_{uv}$ for a given mutation is calculated based on the differential squared Euclidean distance to these prototypes:


(5) $$S_{uv}=∥z_{uv}-p_{\mathrm{passenger}}{∥}^{2}-∥z_{uv}-p_{\mathrm{driver}}{∥}^{2}$$

Through this optimization mechanism, the model is guided to pull driver events toward $p_{\mathrm{driver}}$ while pushing them away from $p_{\mathrm{passenger}}$. Consequently, a higher score indicates that the mutation event lies closer to the driver prototype and further from the passenger prototype in the latent space. EPIC treats these gene-level labels as weak supervision for its event-level prediction tasks. By integrating patient-specific contexts, the model differentiates identical mutations across patients, effectively mitigating the label noise inherent in gene-level catalogs.

### 2.5 Optimization via uncertainty-aware dynamic weighting

To train EPIC robustly, we optimize a composite objective function that balances classification accuracy with information constraints. The primary classification objective is minimized using focal loss ($L_{\mathrm{focal}}$) to handle the inherent class imbalance between driver and passenger mutations. In parallel, to enforce the information constraints, we introduce two regularization terms on the extracted information flows: variance loss ($L_{\mathrm{var}}$), which maximizes the variance of flow norms to prevent feature collapse, and diversity loss ($L_{\mathrm{div}}$), which minimizes the cosine similarity between flow vectors to ensure diverse feature representations.

Instead of manually tuning the hyperparameters for these conflicting objectives, we leverage an uncertainty-based dynamic weighting scheme (Kendall et al. 2018). The total loss function is defined as:


(6) $$L_{\mathrm{total}}=\sum_{k\in \{\mathrm{focal},\mathrm{var},\mathrm{div}\}}\left(\frac{1}{2\sigma_{k}^{2}}L_{k}+\text{log}\;\sigma_{k}\right)$$

where $\sigma_{k}$ represents the learnable homoscedastic uncertainty parameter for each task. This formulation allows the model to adaptively adjust the importance of classification, variance, and diversity objectives during the training process, leading to more stable and optimal convergence.

### 2.6 Implementation details

EPIC was implemented using PyTorch 1.9.1 and PyTorch Geometric 2.0.4. Gene and patient nodes are initialized with 64-dimensional learnable embeddings, projected to a hidden dimension d=128 via linear encoders, and processed by two heterogeneous GATv2 layers with four attention heads. The model is optimized jointly (including prototypes p and uncertainties $\sigma_{k}$) using Adam (learning rate $1\times 10^{-4}$, weight decay $1\times 10^{-5}$) for 1000 epochs, with Focal Loss (α=0.75,γ=2.0) addressing driver event sparsity. All learnable parameters, including the prototype vectors p and the uncertainty parameters $\sigma_{k}$, are jointly optimized through backpropagation. As a transductive network prioritization algorithm, EPIC simultaneously optimizes the entire cohort’s integrated graph to leverage the global topological context.

## 3 Experiments and results

### 3.1 Comparative evaluations

#### 3.1.1 Evaluation metrics

Given the lack of experimentally verified driver labels for individual patients, we utilized cancer-type-specific driver gene lists from the Network of Cancer Genes (NCG 6.0) (Repana et al. 2019) as the pseudo-ground truth. While this benchmark is inherently conservative and may penalize the discovery of novel drivers not yet cataloged, it provides a rigorous standard for assessing personalized accuracy in the absence of individualized gold standards. We adopted a two-track evaluation strategy to validate the model’s effectiveness at both the population and individual levels:

- **Cohort-level evaluation:** This track assesses the model’s ability to capture general oncogenic signals. Individual rankings were aggregated using the Condorcet voting method (Pihur et al. 2008) to generate a population-level priority list, which was then compared against the comprehensive set of known drivers.
- **Individual-level evaluation:** This track measures clinical utility for precision medicine. Metrics were calculated independently for each patient based on their specific mutation profile and averaged across the cohort. Crucially, recall was normalized by the number of known drivers actually present in the patient’s mutations, ensuring a fair assessment of personalized accuracy.

Detailed mathematical definitions of precision, recall, and F1-score for both tracks are provided in Supplementary 1, available as supplementary data at *Bioinformatics* online.

#### 3.1.2 Performance comparison at the cohort level

We evaluated EPIC against six established methods: (i) network-based (PRODIGY and DawnRank), (ii) matrix completion (IMCDriver and PersonaDrive), and (iii) hypergraph-based (PDRWH and PCoDG). For a fair comparison, all methods utilized the identical multi-omics datasets (somatic mutations, gene expression, and PPI network) detailed in the *Data resources and preprocessing* section. Each baseline was executed using its native input requirements and optimal hyperparameters as recommended in its original publication.

**Figure 2:**
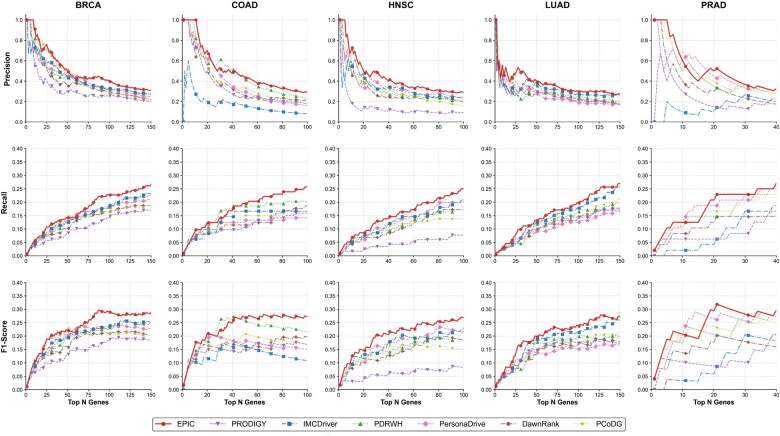
Performance comparison of driver gene identification at the cohort level. The curves illustrate precision, recall, and F1-score across varying cutoffs (Top-*N*) for five cancer types. EPIC consistently outperforms six established baseline methods across all metrics. Notably, the model maintains superior precision in the early rankings and demonstrates robust recall convergence, validating its effectiveness in capturing population-wide oncogenic signals against the background of passenger mutations.

As illustrated in Fig. 2, EPIC (represented by the red solid line) consistently demonstrates superior performance across all metrics and cancer types. A key observation is EPIC’s dominance in precision during the early ranking phase. Across the datasets, particularly in COAD, HNSC, and PRAD, EPIC maintains near-perfect precision at the top of the list, significantly outperforming comparative baselines. This indicates that the event prototyping mechanism successfully maps the most salient driver events to the highest priority scores, minimizing false positives in the critical top ranks.

Furthermore, in terms of recall and F1-score, EPIC exhibits a steady and robust performance curve throughout the entire Top-*N* range. While baseline methods often show slower convergence or instability in capturing the full set of drivers, EPIC consistently achieves the highest sensitivity. This overall superiority suggests that the proposed information-constrained graph learning strategy effectively mitigates the over-smoothing problem common in GNNs, thereby preserving comprehensive driver signals across diverse cancer populations without compromising generalizability.

#### 3.1.3 Performance comparison at the individual level

To demonstrate the practical clinical utility of EPIC, we analyzed the average prediction accuracy for individual patients within the critical Top-1 to Top-5 window. This range is essential for clinicians who require a focused list of high-confidence targets for validation.

**Figure 3:**
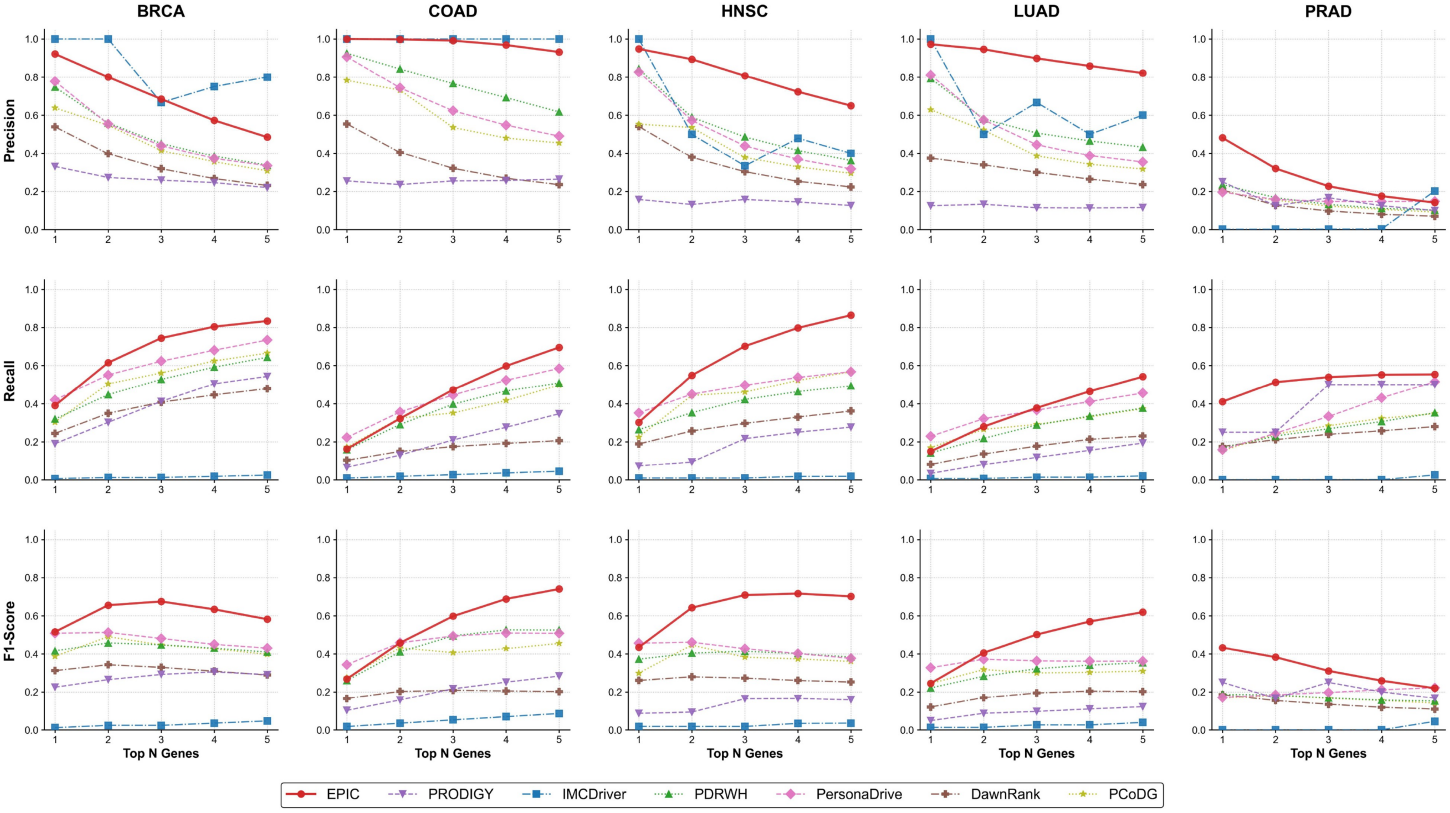
Evaluation of personalized prediction performance at the individual level. The charts display the average metrics for the top-1 to top-5 predicted genes per patient, representing a clinically relevant validation window. EPIC demonstrates exceptional accuracy (Top-1 precision >0.9 in BRCA, COAD, HNSC, LUAD) and exhibits distinct robustness in the heterogeneous PRAD cohort, where it comprehensively outperforms baselines by effectively identifying rare, patient-specific drivers.

As shown in Fig. 3, EPIC comprehensively outperforms baseline methods in both precision and recall. For BRCA, COAD, HNSC, and LUAD, EPIC achieves an average Top-1 precision exceeding 0.9, ensuring that the model’s primary recommendation is a verified driver gene in nearly all cases. The recall curves also exhibit a steep trajectory, capturing the majority of patient-specific drivers within five recommendations, a marked improvement over network-based baselines. To validate these performance gains, we conducted a Wilcoxon signed-rank test. EPIC demonstrated statistically significant improvements over the baselines at the Top-5 threshold across the cohorts (Supplementary 2, Table 1, available as supplementary data at *Bioinformatics* online), confirming its robustness against high patient-to-patient variability.

The robustness of EPIC is most distinct in the challenging PRAD cohort. While matrix completion-based methods like IMCDriver yield negligible scores due to their inherent bias toward reconstructing global population trends rather than identifying specific mutations in an individual’s profile ($M_{k}$), EPIC successfully differentiates rare, individual drivers from background noise. Specifically, EPIC achieves an F1-score exceeding 0.4 at Top-1 in PRAD, significantly surpassing other network-based models which hover around 0.2. This confirms that integrating patient-specific transcriptomic contexts allows EPIC to provide actionable personalized insights even in cohorts with high heterogeneity.

### 3.2 Validation of learning mechanism

To ensure the robustness of our framework, we conducted extensive validation studies regarding the learned metric space and the effectiveness of the information-constrained strategy. First, we visualized the event embedding space, confirming that EPIC successfully maps driver and passenger events into distinct clusters relative to their prototypes (see Supplementary 3, Fig. 1, available as supplementary data at *Bioinformatics* online). Second, we performed an ablation study on network depth. The results demonstrate that our information flow mechanism effectively mitigates the over-smoothing problem, maintaining high representation stability and predictive performance even in deeper architectures, whereas standard GNNs degrade significantly (see Supplementary 4, Fig. 2, available as supplementary data at *Bioinformatics* online). Third, we quantitatively evaluated the individual contributions of the geometric constraints and the uncertainty-based dynamic weighting strategy. An ablation study confirmed that both the geometric constraints (variance and diversity) and the uncertainty-based dynamic weighting are quantitatively essential; removing any of them caused performance drops in deep learning architectures (see Supplementary 5, available as supplementary data at *Bioinformatics* online). Finally, we validated robustness against topological noise, edge attributes, and computational scalability. Despite massive noise from relaxed PPI thresholds, EPIC maintained stable performance and high efficiency. Neutralizing gene expression caused only minor decreases, showing EPIC’s reliance on macroscopic bipartite topology for precision even without quality transcriptomic data (see Supplementary 6, available as supplementary data at *Bioinformatics* online).

### 3.3 Analysis of predicted driver characteristics

To verify that EPIC achieves a robust balance between capturing population-level oncogenic trends and identifying patient-specific rare variants, we analyzed the distributional characteristics of the predicted driver genes. Specifically, we investigated two key aspects: (1) the correlation between model predictions and global mutation frequencies, and (2) the proportion of low-frequency mutations within the top-ranked predictions.

#### 3.3.1 Correlation with mutational landscapes

A prerequisite for a reliable driver prediction model is the ability to recognize well-established, high-frequency driver genes. To validate this, we examined the relationship between a gene’s actual mutational frequency observed in the patient cohort and its predicted recurrence frequency within EPIC’s top-10 outputs, evaluated directly across all genes without prior filtering.

**Figure 4:**
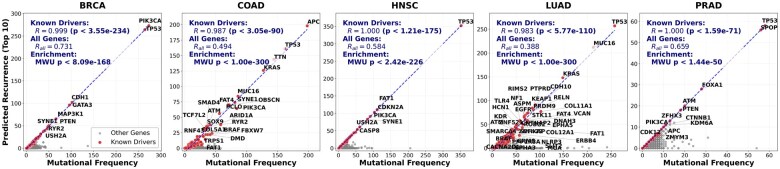
Correlation between mutational frequency (*x*-axis) and predicted driver recurrence (*y*-axis) across five cohorts. High Pearson correlations (*R*) for known drivers (red) confirm population-level statistical capture, while lower overall correlations ($R_{\mathrm{all}}$) and significant Mann–Whitney *U*-tests (MWU *P*) demonstrate the effective suppression of high-frequency passenger mutations (gray).

As shown in Fig. 4, the Pearson correlation coefficients (*R*) computed exclusively for known drivers are exceptionally high (R>0.98) across all five cohorts. Specifically, in BRCA, major biomarkers such as *PIK3CA* and *TP53* are positioned at the top-right, while subtype-specific drivers such as *GATA3* and *CDH1* align perfectly with the regression line, indicating precise recognition of breast cancer etiology (TCGA Network 2012). Similarly, essential targets such as *APC* in COAD (TCGA Network 2012), *KRAS* and *EGFR* in LUAD (TCGA Research Network 2014), and *SPOP* in PRAD (Abeshouse et al. 2015) follow this linear trend. Furthermore, extending this correlation analysis across all genes reveals a significant drop in the overall correlation ($R_{\mathrm{all}}=0.388∼0.731$). This demonstrates that high-frequency passenger mutations (gray dots) are successfully suppressed rather than blindly prioritized. Additionally, a one-sided Mann–Whitney *U*-test confirms that known drivers receive significantly higher prediction scores compared to the background of passenger mutations ($P<10^{-50}$). These results confirm that EPIC balances population-level statistical learning with personalized detection without merely memorizing mutational frequencies.

#### 3.3.2 Identification of low-frequency drivers

While identifying common drivers is a fundamental requirement, the distinct advantage of precision oncology lies in detecting rare, patient-specific driver events (tail mutations) that are often overlooked by population-based frequency analyses. To evaluate this capability, we categorized all genes into three groups based on their mutation frequency within the cohort: high (>10%), medium (5−10%), and low (<5%). We then analyzed the composition of the top-10 predicted driver genes for each patient across the five cancer types.

**Figure 5:**
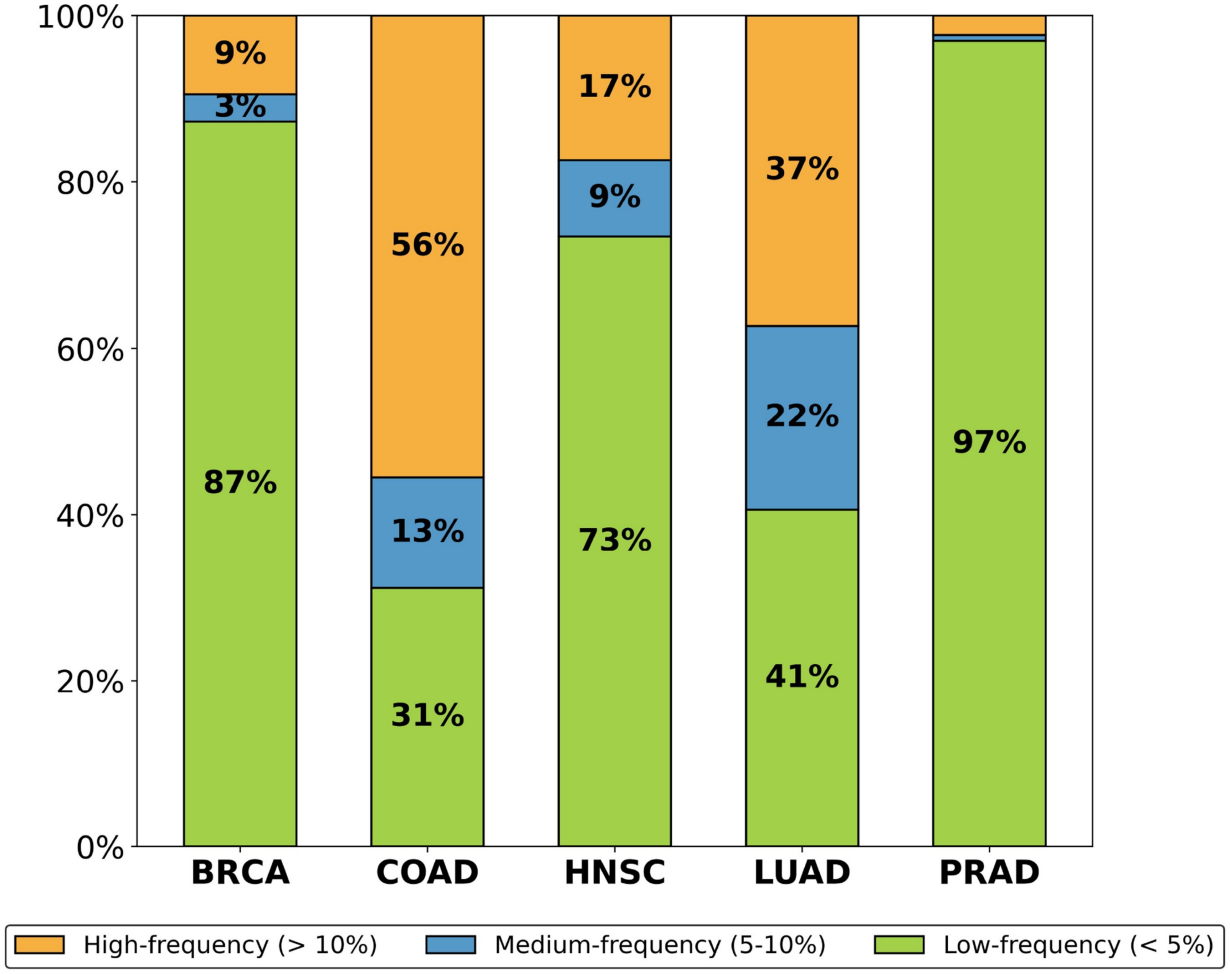
Distribution of mutation frequencies for the top-10 driver genes predicted by EPIC. The vast majority of predictions in BRCA, HNSC, and PRAD are low-frequency variants (<5%), demonstrating the model’s ability to uncover rare, patient-specific drivers beyond common recurrent mutations.

As illustrated in Fig. 5, EPIC prioritizes a substantial proportion of low-frequency variants. This indicates that for the majority of patients, EPIC looks beyond the obvious, highly recurrent genes to identify potential drivers unique to their specific genomic context. In the BRCA, HNSC, and PRAD cohorts, rare driver candidates (<5%) constitute approximately 87%, 73%, and 97% of the predictions, respectively. Notably, the near-total reliance on rare variants in PRAD aligns with its sparse, long-tail mutational landscape (Armenia et al. 2018). EPIC effectively navigates this sparsity by redefining driver prediction as a metric learning task in an event embedding space. By imposing explicit geometric constraints to prevent feature collapse, the model ensures that these subtle, patient-specific driver signals are distinctively preserved rather than being obscured by dominant global trends. This ability to identify rare variants is also evident in COAD and LUAD; despite the prevalence of common drivers such as *APC*, *TP53*, and *KRAS*, 31% and 41% of EPIC’s predictions still belong to the low-frequency category, ensuring that rare co-occurring drivers are not masked by dominant signals.

Collectively, these results demonstrate that EPIC achieves a critical balance between recognizing established oncogenic baselines and discovering personalized rare variants. By reliably capturing statistically significant drivers while simultaneously prioritizing high-confidence rare mutations tailored to the individual patient, the model provides a more comprehensive and clinically relevant landscape of driver genes than traditional frequency-based methods.

#### 3.3.3 Functional enrichment of rare driver candidates

**Figure 6:**
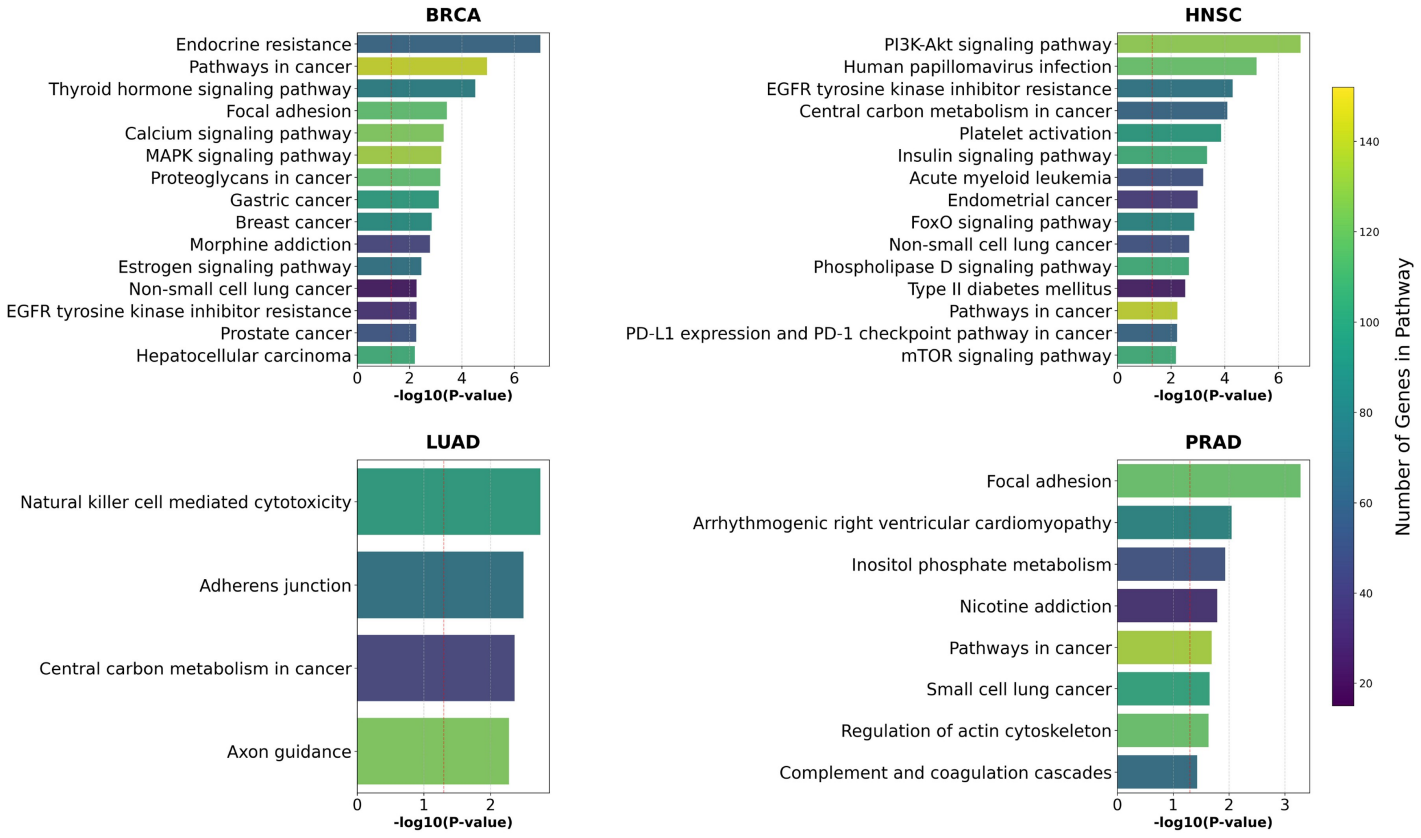
Functional enrichment analysis of predicted low-frequency driver candidates (<5% mutation frequency). The bar charts display the top significantly enriched KEGG pathways for BRCA, HNSC, LUAD, and PRAD. The *x*-axis represents the statistical significance ($-{\;\text{log}\;}_{10}(P-\text{value})$), and the color gradient indicates the number of predicted genes mapped to each pathway. The vertical dashed line indicates the significance threshold (P=0.05). The results demonstrate that low-frequency variants prioritized by EPIC are mapped to biologically critical mechanisms, such as *Endocrine resistance* in BRCA and *Focal adhesion* in PRAD, confirming they are functional drivers rather than random noise.

To verify the biological relevance of the low-frequency drivers (mutated in <5% of the cohort) prioritized by EPIC, we conducted a functional enrichment analysis using the g: Profiler toolkit. Specifically, we mapped these rare candidates to biological pathways within the KEGG database for *Homo sapiens*, identifying significantly enriched pathways with an adjusted *P*-value threshold of .05. As visualized in Fig. 6, these rare candidates are not stochastic noise but are significantly mapped to critical oncogenic signaling cascades tailored to the specific pathology of each cancer type. As a baseline validation, the analysis consistently identified broad oncogenic categories such as *Pathways in cancer* and disease-specific maps (e.g. *Breast cancer*, *Prostate cancer*) across the cohorts, confirming the fundamental biological coherence of the model’s predictions. Beyond these general validations, the analysis revealed mechanistic insights into disease progression.

In BRCA, significant enrichment was observed in *Endocrine resistance* ($P<10^{-7}$) and the *Estrogen signaling pathway*. This aligns with clinical challenges where resistance to hormone therapy constitutes a primary cause of treatment failure, suggesting that EPIC prioritizes patient-specific candidates potentially associated with therapeutic resistance (Ma et al. 2015). For HNSC, the analysis highlighted the *PI3K-Akt signaling pathway* ($P<10^{-7}$) and *Human papillomavirus (HPV) infection*. By capturing the central oncogenic hub (PI3K-Akt) and viral etiologies, the enrichment results are consistent with molecular processes linked to tumor proliferation and viral tumorigenesis (Lui et al. 2013, TheCancerGenomeAtlasNetwork 2015). In LUAD, the identified pathways, including *Natural killer (NK) cell mediated cytotoxicity* (P<0.002) and *Adherens junction* (P<0.004), are associated with mechanisms of immune evasion and invasion. These results suggest that EPIC detects EPIC detects rare candidates potentially involved in processes related to immune surveillance escape and the initiation of epithelial-to-mesenchymal transition (EMT) (Malladi et al. 2016). Finally, in PRAD, pathways regulating structural dynamics such as *Focal adhesion* ($P<10^{-3}$) and *Regulation of actin cytoskeleton* were predominant. Given the high propensity of prostate cancer for bone metastasis, the prioritization of these cell motility-related pathways suggests that the model captures signals relevant to the metastatic potential of the tumor (Gandaglia et al. 2014, Robert et al. 2020). Furthermore, for COAD, due to its highly fragmented mutational landscape, functional enrichment was evaluated through a separate, tailored analysis (an expanded <10% threshold with recurrence-based denoising) to overcome extreme signal sparsity. This modified approach revealed the enrichment of individualized rare candidates in critical cascades, such as the *TGF-beta signaling pathway* (detailed in Supplementary 7, Fig. 4, available as supplementary data at *Bioinformatics* online).

Overall, these findings validate that the low-frequency genes identified by EPIC are functional drivers associated with specific pathogenic mechanisms, rather than representing stochastic noise. To rigorously validate that these signals are not artifacts of general mutation frequencies, we compared our results against frequency-matched random gene sets, confirming that EPIC’s predictions yield significantly stronger enrichment signals (detailed in Supplementary 8, available as supplementary data at *Bioinformatics* online). Notably, several top-ranked candidates predicted by the model are not included in the cancer-type-specific NCG ground truth. However, these candidates exhibit high pathway coherence and literature-supported oncogenic roles, confirming EPIC’s potential for uncovering novel regulatory mechanisms beyond established knowledge (Supplementary 9, available as supplementary data at *Bioinformatics* online).

### 3.4 Expansion of clinical actionability

To evaluate the translational potential of EPIC in precision oncology, we quantified its ability to expand the population of patients eligible for targeted therapies. Specifically, we assessed whether the model could identify actionable driver genes in patients who lacked therapeutic options when relying solely on known driver lists.

To ensure the clinical validity of our analysis, we curated a high-confidence list of actionable genes by integrating three premier databases: Precision Oncology Knowledge Base (OncoKB) (Chakravarty et al. 2017), Clinical Interpretation of Variants in Cancer (CIViC) (Griffith et al. 2017), and Drug Gene Interaction Database (DGIdb) (Freshour et al. 2021). We strictly filtered these sources for “Oncology” and “Clinical Utility.” For OncoKB, we selected genes classified under Therapeutic Levels 1–4, resulting in 87 high-evidence targets. From CIViC, we included entries with predictive evidence levels A (Proven), B (Clinical Trial), and C (Case Study), yielding 242 genes. Finally, for DGIdb, we applied an intersection strategy, retaining only 461 genes that are both clinically actionable and interact with anti-neoplastic or immunotherapeutic drugs.

We defined *Baseline Coverage* as the percentage of patients harboring at least one actionable mutation within the set of known driver genes. To quantify the additional therapeutic value provided by our framework, we measured the *Expanded Coverage*. This metric denotes the percentage of patients for whom EPIC’s top-10 predictions newly identified actionable targets that were missed by known driver lists.

**Figure 7:**
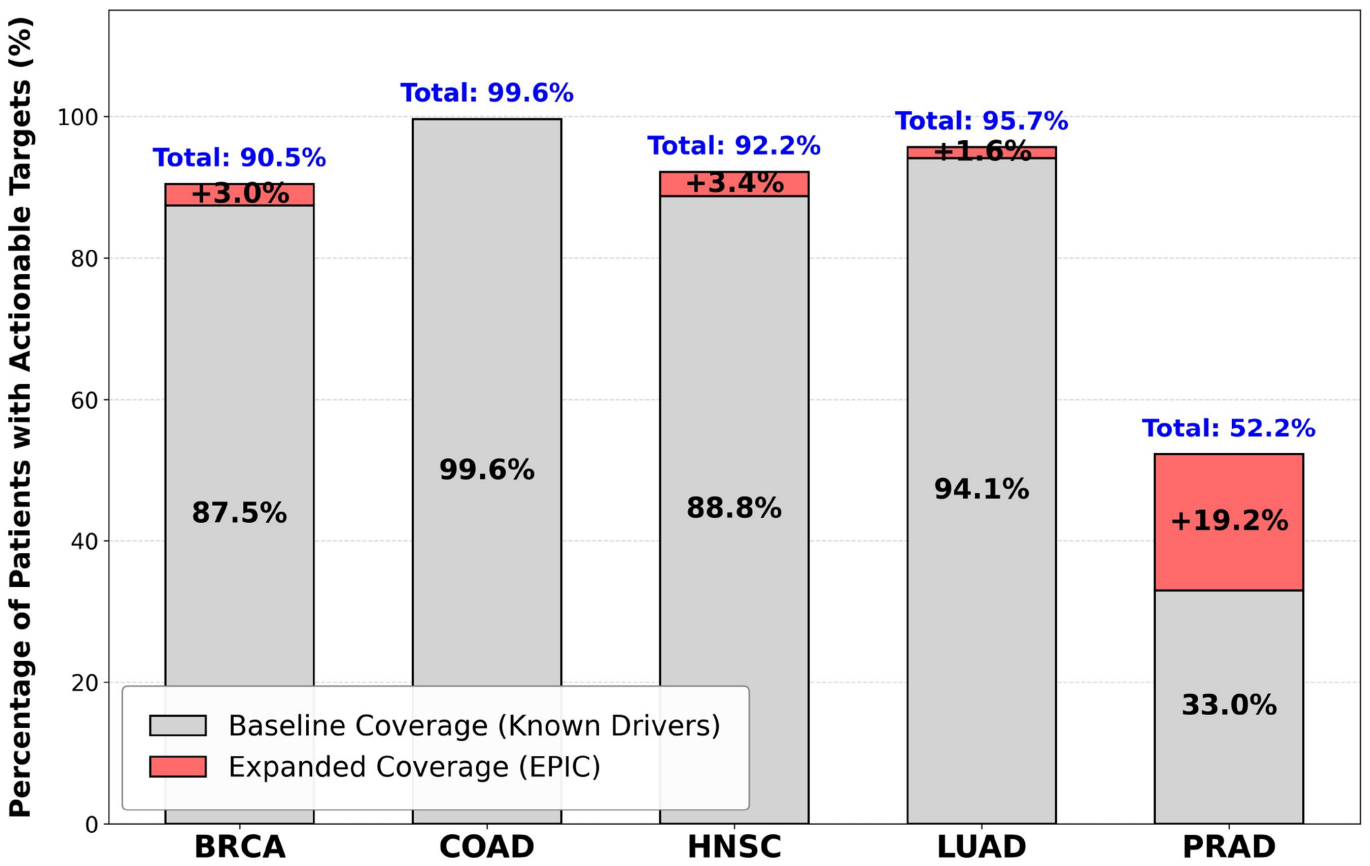
Expansion of the clinically actionable patient population by EPIC. Baseline coverage represents the proportion of patients covered by known drivers, whereas additional coverage indicates the proportion of patients for whom EPIC identified previously unrecognized actionable targets. EPIC expanded therapeutic opportunities in most cohorts, effectively identifying actionable targets for patients previously considered target-negative.

As shown in Fig. 7, EPIC successfully expanded the therapeutic horizon in four out of five cancer cohorts. The most striking impact was observed in PRAD, where EPIC identified actionable targets for an additional 19.2% of patients, raising the total coverage from 33.0% to 52.2%. Although COAD remained at its near-saturated 99.6%, EPIC successfully extended potential therapeutic options in other cohorts with high baseline coverage (>87%), such as BRCA, HNSC, and LUAD, by an additional 3.0%, 3.4%, and 1.6%, respectively. These findings indicate that EPIC effectively identifies actionable drivers in the “long tail” of mutation distributions, bridging the gap between genomic data and therapeutic decision-making for patients falling outside standard treatment guidelines.

## 4 Discussion and conclusion

In this study, we proposed EPIC, an event-centric graph learning framework for personalized cancer driver discovery. EPIC addresses the critical challenge of driver signal dilution by integrating event prototyping with an information-constrained strategy. By imposing explicit geometric constraints, our model effectively prevents feature collapse and GNN over-smoothing, ensuring that rare driver signals are preserved and distinguished from dominant passenger noise. Our results demonstrate superior biological insight and clinical utility, successfully prioritizing long-tail variants associated with key mechanisms like endocrine resistance. Notably, EPIC identified additional actionable targets for PRAD patients previously considered target-negative. Crucially, many top-ranked candidates outside existing benchmarks exhibit high biological plausibility, indicating EPIC’s potential to uncover novel regulatory mechanisms beyond established knowledge.

Despite these strengths, we acknowledge the potential and fundamental limitations regarding gene-length bias, where long genes may accumulate stochastic mutations independently of their oncogenic role. Additionally, the current framework relies on a single biological network. While our Correlation with Mutational Landscapes analysis suggests a degree of inherent resistance to such artifacts, future iterations will explicitly incorporate heterogeneous network fusion (e.g. regulatory graphs), background mutation rate (BMR) models, and multi-omics data (e.g. CNVs) to further refine specificity. Ultimately, we aim to provide a comprehensive solution for personalized driver discovery, bridging the gap between computational genomics and actionable precision medicine.

## Author contributions

Sang-Pil Cho (Conceptualization, Methodology, Software, Formal analysis, Investigation, Data curation, Writing—original draft, Writing—review & editing, Visualization), and Young-Rae Cho (Conceptualization, Methodology, Resources, Writing—review & editing, Supervision, Project administration, Funding acquisition)

## Supplementary Material

btag229_Supplementary_Data

## Data Availability

The source codes and datasets used in this study are publicly accessible at https://github.com/spcho-dev/EPIC.
