# Supplementary material for: EPIC: Event Prototyping via Information Constrained graph learning for personalized cancer driver gene prediction
Source: Bioinformatics. 2026 Jul 7;42(Suppl 1):btag229. doi: 10.1093/bioinformatics/btag229 (PMC13340217; doi:10.1093/bioinformatics/btag229)
Supplement: btag229_Supplementary_Data [file btag229_supplementary_data.pdf]

Supplementary materials for:

EPIC: Event Prototyping via Information Constrained graph learning for personalized cancer driver gene prediction

Sang-Pil Cho[1] and Young-Rae Cho[1,2,*]

[1]Division of Software, Yonsei University Mirae Campus, 1 Yeonsedae-gil, 26493, Gangwon-do, Republic of Korea

[2]Division of Digital Healthcare, Yonsei University Mirae Campus, 1 Yeonsedae-gil, 26493, Gangwon-do, Republic of Korea

*Corresponding author: youngcho@yonsei.ac.kr

Supplementary 1. Definition of Evaluation Metrics

Personalized cancer driver gene prediction is formulated as a ranking problem rather than a simple binary classification task. Since the true driver genes driving tumorigenesis in specific individual patients remain largely unknown, we utilized cancer-type-specific driver gene lists from the Network of Cancer Genes (NCG 6.0) as the ground truth for evaluation. To rigorously validate the model under this uncertainty, we adopted a Two-Track evaluation strategy assessing effectiveness at both the population and individual levels.

1. **Cohort-level Evaluation Metrics**
   The cohort-level evaluation assesses the model's ability to capture general oncogenic signals across a specific cancer population. To perform this, we first aggregated the individualized rankings of all patients within a cohort using the Condorcet voting method to generate a single population-level priority list. The metrics were then calculated by comparing the top N genes of this aggregated list against the entire set of cancer-type-specific drivers defined in NCG 6.0.

   Let $\mathrm{C_{agg}}[N]$ be the set of top N genes from the aggregated priority list and D_{all} be the total set of known driver genes for the given cancer type. The cohort-level metrics are defined as:

$$Precision_{cohort} = \frac{|C_{\mathrm{agg}}[N] \cap D_{all}|}{N}, \qquad Recall_{cohort} = \frac{|C_{agg}[N] \cap D_{all}|}{|D_{all}|}$$

2. **Individual-level Evaluation Metrics**
   The individual-level evaluation measures the clinical utility for precision medicine by verifying how accurately the model identifies driver mutations within a specific patient's unique mutational profile. In this track, metrics are calculated for each patient independently based on their specific mutation set M_k and then averaged across the entire cohort of K patients.

   Crucially, the denominator for Recall is restricted to the known driver genes actually present in that specific patient's mutations, ensuring a fair assessment of personalized prediction accuracy. Let $\mathrm{C}_k[N]$ be the top N predicted genes for patient k, and R_k be the set of ground-truth driver genes present in that patient's profile (defined as $R_k = M_k \cap D_{all}$). The individualized metrics are calculated as follows:

$$Precision_{cohort} = \frac{1}{K}\sum_{k=1}^{K}\frac{|C_k[N] \cap R_k|}{N}, \qquad Recall_{cohort} = \frac{1}{K}\sum_{k=1}^{K}\frac{|C_k[N] \cap R_k|}{|R_k|}$$

3. **F1-Score**
   For both tracks, the F1-Score is calculated as the harmonic mean of the respective Precision and Recall values:

$$F1 - Score = 2 \cdot \frac{Precision \cdot Recall}{Precision + Recall}$$

Supplementary 2. Statistical Validation of Model Performance

In our cohort-level evaluation, individual patient rankings were aggregated into a single consensus list using the Condorcet voting method. Because this aggregation produces only a single deterministic metric score for the entire cohort, it inherently lacks the sample variance required for statistical testing. Therefore, to rigorously validate the statistical significance of EPIC's performance improvements, we focused on the individual-level evaluation. This approach not only provides a sufficient sample size (i.e., the number of patients in each cohort) but also rigorously tests the model's robustness against high patient-to-patient variability, which is the primary objective of personalized precision oncology.

We conducted a one-sided Wilcoxon signed-rank test comparing the patient-wise Precision and Recall scores of EPIC against each baseline method at the Top-5 prediction threshold. The alternative hypothesis was that the scores generated by EPIC are strictly greater than those of the baseline models.

Table S1. Wilcoxon signed-rank test p-values comparing EPIC against baseline models at the Top-5 threshold..

Cancer Cohort	Baseline Model	Precision P-value (vs. EPIC)	Recall P-value (vs. EPIC)
BRCA	*DawnRank*	1.9536e-92	1.9066e-92
	PRODIGY	7.3572e-16	1.6583e-12
	IMCDriver	1.0000e+00	1.0000e+00
	PersonaDrive	3.9629e-71	5.4665e-71
	PDRWH	7.4656e-59	2.3023e-48
	PCoDG	1.1322e-73	1.6475e-73
COAD	*DawnRank*	1.3176e-46	9.8292e-46
	PRODIGY	2.6539e-21	4.2508e-21
	IMCDriver	1.0000e+00	1.0000e+00
	PersonaDrive	8.5991e-44	4.1385e-43
	PDRWH	2.7109e-39	1.6303e-38
	PCoDG	2.5169e-37	9.5370e-37
HNSC	*DawnRank*	3.9641e-71	4.5716e-71
	PRODIGY	5.6993e-05	5.0264e-05
	IMCDriver	1.0483e-47	7.7324e-17
	PersonaDrive	7.0500e-66	7.7283e-66
	PDRWH	8.3375e-60	6.8827e-52
	PCoDG	7.5258e-65	7.7324e-65
LUAD	*DawnRank*	1.2135e-78	1.8493e-77
	PRODIGY	4.5687e-16	2.5916e-15
	IMCDriver	7.6143e-76	8.6372e-19
	PersonaDrive	3.6439e-77	5.5314e-76
	PDRWH	3.8877e-75	1.0107e-71
	PCoDG	4.5114e-69	3.8062e-68
PRAD	*DawnRank*	1.4257e-27	3.5334e-26
	PRODIGY	5.0162e-17	4.6512e-12
	IMCDriver	2.5024e-16	2.5026e-16
	PersonaDrive	3.5846e-17	1.6814e-16
	PDRWH	6.5670e-18	5.1208e-15
	PCoDG	2.9478e-23	7.2581e-22

As detailed in Table S1, EPIC demonstrated highly significant improvements ($P < 0.05$) across the vast majority of comparisons in all five cancer cohorts. We observed specific exceptions, such as IMCDriver, which highlight an inherent methodological characteristic rather than a performance gap in our model. The fundamental goal of personalized prediction is to identify specific oncogenic drivers unique to an individual patient. However, IMCDriver exhibited a strong population bias by consistently prioritizing the most statistically recurrent genes across the cohort, often overshadowing the distinct transcriptomic context of each individual. When evaluated against the ground truth, this frequency-driven approach can inflate the average precision score simply because these common mutations are highly prevalent across the population. Consequently, this generalized prioritization limits the degree of personalization and results in tied scores that skew the p-value calculation, diverging from the core objective of precision medicine.

Supplementary 3. Visualization of Event Embedding Space

To intuitively verify the classification logic of EPIC, we visualized the distribution of mutation event embeddings relative to the learned prototypes. We calculated the Euclidean distances of all mutation events to both the Driver Prototype (P_{driver}) and Passenger Prototype ($P_{passenger}$).

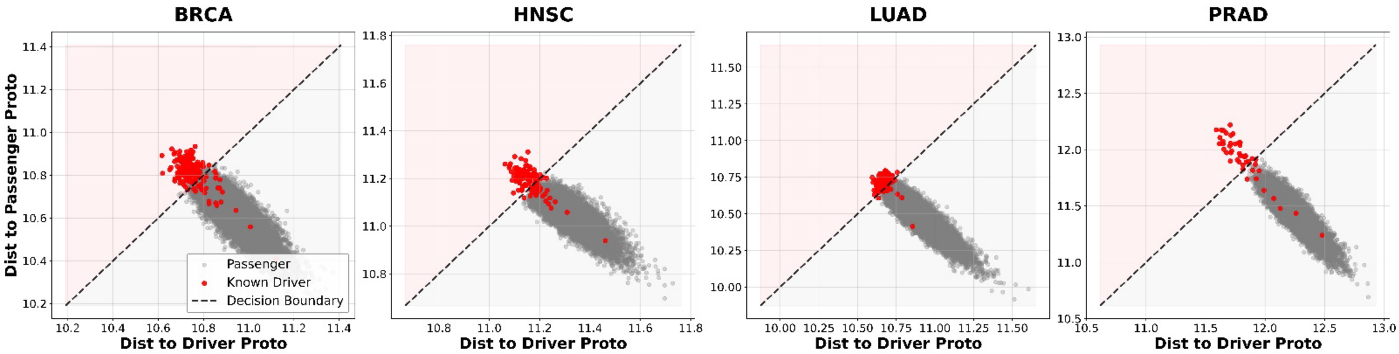

Figure S1. Scatter plot of distances to driver and passenger prototypes. Red dots represent known drivers, and grey dots represent passenger mutations. The dashed line indicates the decision boundary ($y = x$).

Scatter plot of distances to driver and passenger prototypes. Red dots represent known drivers, and grey dots represent passenger mutations. The dashed line indicates the decision boundary ($y = x$). As depicted in Figure S1, known driver events (red dots) are predominantly located above the decision boundary ($y = x$), indicating that their embeddings are significantly closer to the Driver Prototype than to the Passenger Prototype. Conversely, passenger events (grey dots) cluster densely below the boundary. This distinct separation confirms that EPIC successfully learns a discriminative metric space where oncogenic features are mapped in proximity to the ideal driver prototype.

Supplementary 4. Evaluation of Information Preservation against Oversmoothing

A critical limitation in applying deep Graph Neural Networks (GNNs) to biological networks is the "over-smoothing" phenomenon, where node representations become homogenized as the network depth increases. This is particularly detrimental in cancer driver prediction, as subtle, patient-specific driver signals can be easily washed out by dominant topological trends (e.g., common passenger mutations).

To validate that our proposed Information Constrained learning strategy effectively mitigates this issue, we conducted a comparative analysis against a baseline Vanilla GAT model. We evaluated the model's ability to maintain feature distinctiveness and predictive accuracy while varying the network depth from $L = 1$ to $L = 6$ on the BRCA dataset[2].

Metric for Feature Distinctiveness To quantitatively assess the preservation of unique genomic contexts, we utilized the Class Separation Index (CSI). The CSI measures the Euclidean distance between the centroids of the learned embeddings for known driver events (μ_{driver}) and passenger events ($\mu_{passenger}$) in the latent space [3]. The CSI is defined as:

$$CSI = ||\mu_{driver} - \mu_{passenger}||_2$$

A higher CSI indicates that the model successfully imposes geometric constraints to maintain the diversity of feature representations, preventing the embedding space from collapsing into a uniform distribution.

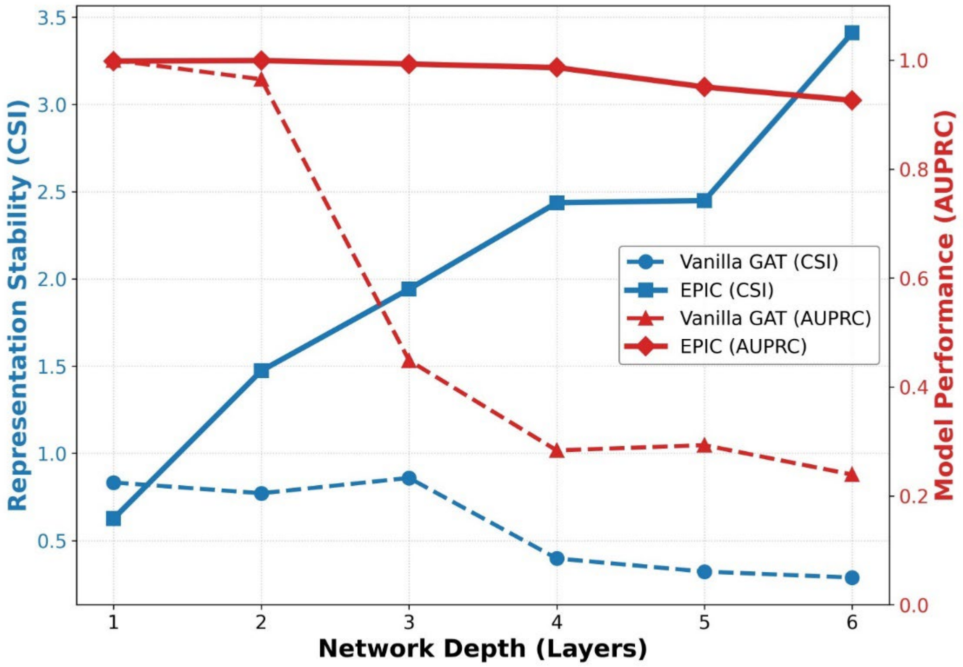

Figure S2. Impact of Information Constrained learning on representation robustness. Comparison of CSI (blue) and AUPRC (red) between EPIC (solid lines) and Vanilla GAT (dashed lines) across varying network depths.

The results, illustrated in Supplementary Figure S2, provide empirical evidence for the efficacy of our strategy. The Vanilla GAT model (dashed lines) exhibits a characteristic degradation in both representation stability (CSI) and model performance (AUPRC) as the network depth increases beyond 3 layers. This confirms that without explicit constraints, the distinct genomic signatures of driver events become indistinguishable from the background noise of passenger mutations.

In contrast, EPIC (solid lines) demonstrates superior robustness in deeper architectures. Notably, the CSI metric for EPIC increases with depth, reaching its peak at 6 layers. This trend suggests that the Information Flow mechanism effectively leverages long-range biological interactions to disentangle rare driver signals from noise, rather than allowing them to degrade. Consequently, the predictive performance (AUPRC) remains consistently high (> 0.9), validating that EPIC successfully preserves biological specificity even in deep graph learning scenarios.

Supplementary 5. Loss Components & Uncertainty Weight Ablation

To rigorously address the contributions of our proposed geometric constraints and the uncertainty-based dynamic weighting strategy, we conducted an in-depth analysis of the learned parameters and a comprehensive ablation study.

Evolution of Uncertainty-based Dynamic Weights

In EPIC, we employ homoscedastic uncertainty (σ^2) to dynamically balance the primary classification task ($\mathcal{L}_{focal}$) and the information constraints ($\mathcal{L}_{var}$ and $\mathcal{L}_{div}$). To understand how the model prioritizes these objectives during training, we tracked the evolution of the dynamic weight values, defined as $0.5 \times exp(-\sigma^2)$, across 1000 epochs.

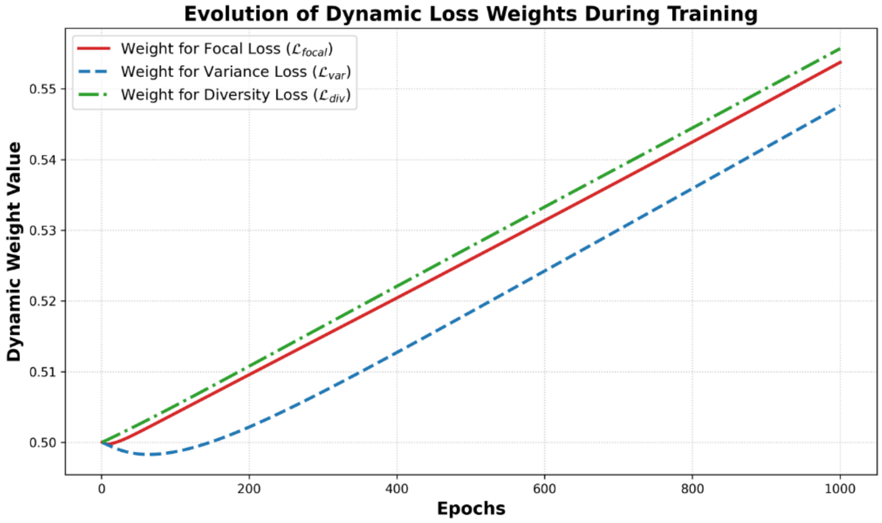

Figure S3. Evolution of dynamic loss weights during training. The plot illustrates the adaptive adjustment of weights for focal loss (red solid line), variance loss (blue dashed line), and diversity loss (green dash-dot line) derived from the learned uncertainty parameters (σ^2).

As shown in Figure S3, all weights initialize equally at 0.50. During the early phases of training, the weight for the variance loss slightly decreases, allowing the model to prioritize the initial classification of obvious driver mutations while establishing basic representation diversity. As training progresses and the network approaches deeper layers where over-smoothing typically occurs, the model automatically increases the weights for both the diversity and variance constraints. This adaptive scaling confirms that the network learns to actively enforce geometric constraints to preserve the unique transcriptomic contexts of rare driver events, rather than allowing them to collapse into a mean field.

Quantitative Ablation of Learning Mechanisms

To directly answer how the model would perform if we simply minimized the unweighted sum of losses (i.e., replacing the dynamic σ^2 weighting with static, uniform weights), and to quantify the individual contributions of the geometric constraints, we evaluated five model variants at the clinically critical Top-5 prediction threshold. All models in this ablation study were configured with a 4-layer architecture ($L = 4$) to intentionally simulate a deep graph learning environment where the risk of over-smoothing is highly pronounced.

Table S2. Ablation Study of information constraints and dynamic weighting at the Top-5 prediction threshold.

Model Variant	$\mathcal{L}_{focal}$	$\mathcal{L}_{var}$ $(Variance)$	$\mathcal{L}_{div}$ $(Diversity)$	Weighting Strategy	Precision@5	Recall@5	F1-Score@5
$EPIC\ (Proposed)$	O	O	O	Dynamic (σ^2)	**0.4879**	**0.8365**	**0.6165**
$EPIC\ (Static)$	O	O	O	Static (e.g., 1.0 for all)	0.4301	0.7801	0.5548
$EPIC\ (w/o\ Var)$	O	X	O	Dynamic	0.3906	0.7507	0.5138
$EPIC\ (w/o\ Div)$	O	O	X	Dynamic	0.3805	0.7405	0.5026
$EPIC\ (w/o\ Base)$	O	X	X	N/A (Only Classification)	0.3102	0.6401	0.4179

As detailed in Table S2, replacing the uncertainty-based dynamic weighting with static weights (w/o Dynamic Weight) results in a degradation in performance. This explicitly demonstrates that simply minimizing the unweighted sum of losses leads to suboptimal convergence, validating the necessity of the σ^2 parameters for dynamic task-balancing.

Furthermore, removing specific geometric constraints severely impaired the model's capacity. The ablation of the variance constraint (w/o Variance Loss) or the diversity constraint (w/o Diversity Loss) independently caused substantial performance drops. The Base model, which relies solely on the focal loss without any information constraints, suffered the most severe decline. This highlights the inherent vulnerability of standard GNNs to over-smoothing and confirms that our proposed constraints are quantitatively essential for accurate, personalized driver gene prediction.

Supplementary 6. Robustness to Graph Construction and Computational Scalability

To ensure that the performance of EPIC is not overly sensitive to graph construction parameters and to evaluate its computational efficiency, we conducted a robustness analysis focusing on the Protein-Protein Interaction (PPI) confidence threshold and the use of gene expression attributes.

A potential concern in deep graph learning is that relying on a stringent PPI confidence score (e.g., $\geq$ 0.85) might introduce a strong bias toward well-studied hub nodes, limiting the discovery of novel or isolated drivers. To investigate this, we relaxed the confidence threshold to 0.7 and 0.4, progressively introducing a massive amount of lower-confidence interactions into the network. Furthermore, we evaluated the impact of utilizing patient-specific gene expression levels as edge attributes In the w/o Expression Attributes variant, which was evaluated using the default PPI > 0.85 network to ensure a fair comparison with our proposed model, we neutralized the transcriptomic context by setting all mutation edge weights to a uniform value of 1.0, preserving only the binary topology of the mutation bipartite graph.

Table S3. Network Statistics across Graph Construction Variants (BRCA Cohort).

Network Construction Variant	$STRING\ \ Network$		$EPIC\ Heterogeneous\ Graph$		
	Nodes	Edges[a]	Total Nodes (*Genes / Patients*)	PPI Edges[b]	Mutation Edges
$PPI\ \geq\ 0.85$ (Proposed)	9,993	242,176	19,405 (18,616 / 789)	460,484	53,584
$PPI\ \geq\ 0.70$	15,163	360,638	19,405 (18,616 / 789)	675,556	53,584
$PPI\ \geq\ 0.40$	16,992	840,100	19,405 (18,616 / 789)	1,559,794	53,584
$w/o\ Expression\ Attributes$	9,993	242,176	19,405 (18,616 / 789)	460,484	53,584

[a] Raw STRING Edges represent the number of unique, undirected protein-protein interactions directly obtained from the STRING database. [b] EPIC PPI Edges represent the number of directed edges (i.e., modeled as both $A \rightarrow B$ and $B \rightarrow A$) constructed for message passing in PyTorch Geometric, after strictly filtering for genes present in the patient multi-omics dataset.

Table S4. Robustness and Scalability evaluation of EPIC at the Top-5 prediction threshold.

Network	Precision@5	Recall@5	F1-Score@5	Avg. Time / Epoch	Total Time (1000 epoch)
$PPI\ \geq\ 0.85$ (Proposed)	0.4879	0.8365	0.6165	117.50 ms	1.96 min
$PPI\ \geq\ 0.70$	0.4877	0.8361	0.6159	702.93 ms	11.72 min
$PPI\ \geq\ 0.40$	0.4874	0.8358	0.6156	5149.09 ms	85.82 min
$w/o\ Expression\ Attributes$	0.4765	0.8210	0.6030	-	-

As detailed in Table S3, lowering the threshold to 0.40 drastically expands the raw PPI network (from 9,993 to 16,922 nodes, and from 242,176 to 840,100 edges). When mapped to the patient-specific multi-omics data, this results in nearly tripling the number of PPI edges propagated through the EPIC framework (from 460,484 to 1,559,794 edges). Despite this massive influx of low-confidence edges and topological noise, EPIC maintained exceptionally stable predictive performance (Table S4). The F1-Score remained nearly identical, demonstrating that our Information Constrained framework effectively filters out topological noise and is highly robust against variations in the underlying interaction network. The stringent 0.85 threshold was selected not to artificially inflate hub biases, but to conservatively ensure the highest biological reliability of the identified pathways.

Neutralizing the expression attributes resulted in a moderate decrease in predictive performance. While this variation confirms that patient-specific transcriptomic context provides valuable and significant signals for accurately identifying personalized drivers, the model still maintains a robust baseline accuracy. This indicates that rather than overfitting to scalar expression values, EPIC derives a substantial portion of its predictive power from the macroscopic bipartite network topology (i.e., the structural connectivity between specific patients and mutated genes) in conjunction with the PPI network. Clinically, this highlights a significant advantage: it demonstrates that EPIC can still deliver highly competitive precision even in challenging scenarios where RNA-seq expression data is unavailable or of poor quality, relying robustly on binary somatic mutation profiles.

From a theoretical perspective, the GATv2-based message-passing in EPIC follows a complexity of $O(V + E)$, and the event-level prototyping scales linearly with the number of observed mutations $O(|E_{mut}|)$. This linear scalability ensures that EPIC is computationally efficient and suitable for large-scale pan-cancer analysis or clinical integration. To provide empirical evidence, we measured the training time on a server equipped with an NVIDIA GeForce RTX 3080 GPU (10GB VRAM). As summarized in Table S4, the default configuration (0.85 threshold) is highly efficient, completing 1,000 epochs in only 1.96 minutes. Notably, even as the network density increases nearly threefold in the 0.40 variant, the runtime remains within a feasible range (85.82 minutes), confirming that the framework can handle massive-scale biological networks without exponential increases in overhead.

Supplementary 7. Collective Functional Enrichment Analysis of Personalized Rare Drivers in COAD

For most cancer cohorts (e.g., BRCA, HNSC, LUAD, PRAD), analyzing rare driver candidates mutated in less than 5% of the population successfully revealed significant functional pathways. However, applying this strict <5% threshold to the COAD cohort resulted in extreme signal sparsity. Because high-frequency mutations (e.g., APC, TP53, KRAS) dominate the primary oncogenic pathways in COAD, the remaining low-frequency predictions <5% were highly individualized and excessively fragmented across hundreds of unique genes. Consequently, a standard aggregate enrichment analysis on this <5% pool failed to achieve statistical significance, as the patient-specific signals were too dispersed across disparate cellular mechanisms.

Expanded Threshold (<10%) and Recurrence-based Denoising

To overcome this COAD-specific limitation and capture the biological consensus of its individualized mutations, we adjusted the rare driver threshold to <10% and employed a recurrence-based denoising strategy. First, to prevent the analysis from being overshadowed by the globally dominant drivers, we explicitly decoupled the signals by strictly excluding all high-frequency variants (>10%) from the query. Second, from the expanded rare driver pool (<10%), we ranked the candidates based on their prediction recurrence across the patient population. We then selected the top 100 "elite" genes—those that are demographically uncommon yet repeatedly and confidently identified by EPIC as personalized drivers across multiple patients. This filtering acts as a powerful signal amplifier, isolating functionally critical rare variants from stochastic background noise.

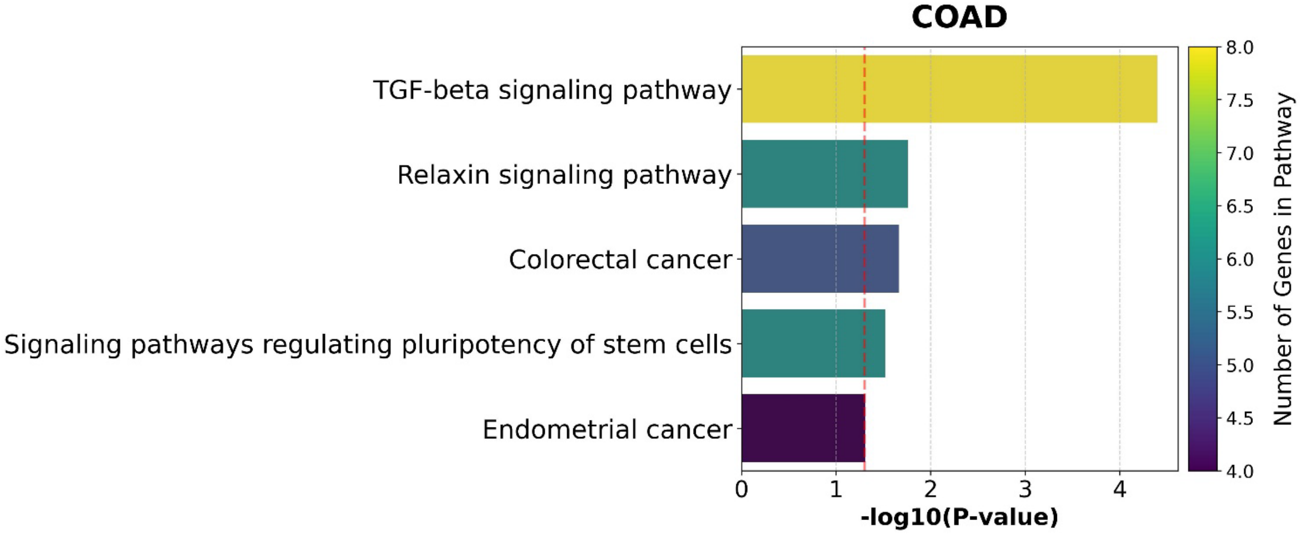

Figure S4. Functional enrichment of recurrent low-frequency drivers in COAD. Significantly enriched KEGG pathways for the top 100 personalized rare drivers (<10% mutation frequency), selected via a recurrence-based denoising strategy. The x-axis shows statistical significance ($-log_{10}(P - value)$), color indicates the number of mapped genes, and the red dashed line marks P=0.05. These results suggest that highly individualized mutations may collectively be associated with critical disease cascades, such as EMT and tumor microenvironment remodeling.

As illustrated in Figure S4, the g:Profiler analysis of these top 100 recurrent rare drivers revealed enrichment in several oncogenic cascades. The most significantly enriched pathway was the TGF-beta signaling pathway. This is biologically relevant, as TGF-beta signaling is a known axis for Epithelial-to-Mesenchymal Transition (EMT) and metastasis in colorectal cancer, which can contribute to late-stage progression alongside the globally prevalent standard cascades [1]. Furthermore, the enrichment of the Relaxin signaling pathway suggests a potential connection to auxiliary mechanisms associated with cancer cell motility, angiogenesis, and tissue remodeling in the tumor microenvironment [2]. Additionally, the term Signaling pathways regulating pluripotency of stem cells is consistent with processes involved in the maintenance of cancer stem cell populations and therapeutic resistance in colorectal tumors [3].

These results suggest that EPIC's rare, patient-specific predictions in COAD likely reflect underlying biological processes rather than random computational artifacts. By tailoring the threshold to the cohort's specific landscape and applying recurrence-based denoising, the analysis indicates that these individualized mutations may collectively be associated with critical hallmarks of cancer.

References for Supplementary 7

[1] Itatani, Yoshiro, Kenji Kawada, and Yoshiharu Sakai. "Transforming growth factor-β signaling pathway in colorectal cancer and its tumor microenvironment." *International journal of molecular sciences* 20.23 (2019): 5822.

[2] Jung, Jungchan, and Hyunho Han. "The diverse influences of relaxin-like peptide family on tumor progression: Potential opportunities and emerging challenges." *Heliyon* 10.2 (2024).

[3] Vermeulen, Louis, et al. "Wnt activity defines colon cancer stem cells and is regulated by the microenvironment." *Nature cell biology* 12.5 (2010): 468-476.

Supplementary 8. Biological Plausibility of Predicted Novel Driver Candidates

To rigorously evaluate whether the functional enrichment observed in EPIC's rare driver candidates exceeds what might be expected by chance, we performed a random permutation test (N=100 iterations) for each cancer cohort.

Background Pool Construction: For each cohort, a background gene pool was constructed containing all genes with a population mutation frequency below the specified threshold (<5% for BRCA, HNSC, LUAD, PRAD; <10% for COAD).

Null Model Generation: In each iteration, we randomly sampled a gene set from the background pool. The size of this random set was matched to the number of rare driver candidates prioritized by EPIC for that specific cohort (e.g., Top 100 refined candidates for COAD).

Comparative Pathway Analysis: Each random gene set was subjected to functional enrichment analysis using g:Profiler (KEGG source, adjusted P < 0.05). The number of significantly enriched pathways was recorded to establish a null distribution of functional signals inherent in low-frequency variants.

Empirical P-value Calculation: The empirical P-value was defined as the proportion of iterations where the random gene set yielded a number of significant pathways equal to or greater than that of the EPIC-prioritized candidates.

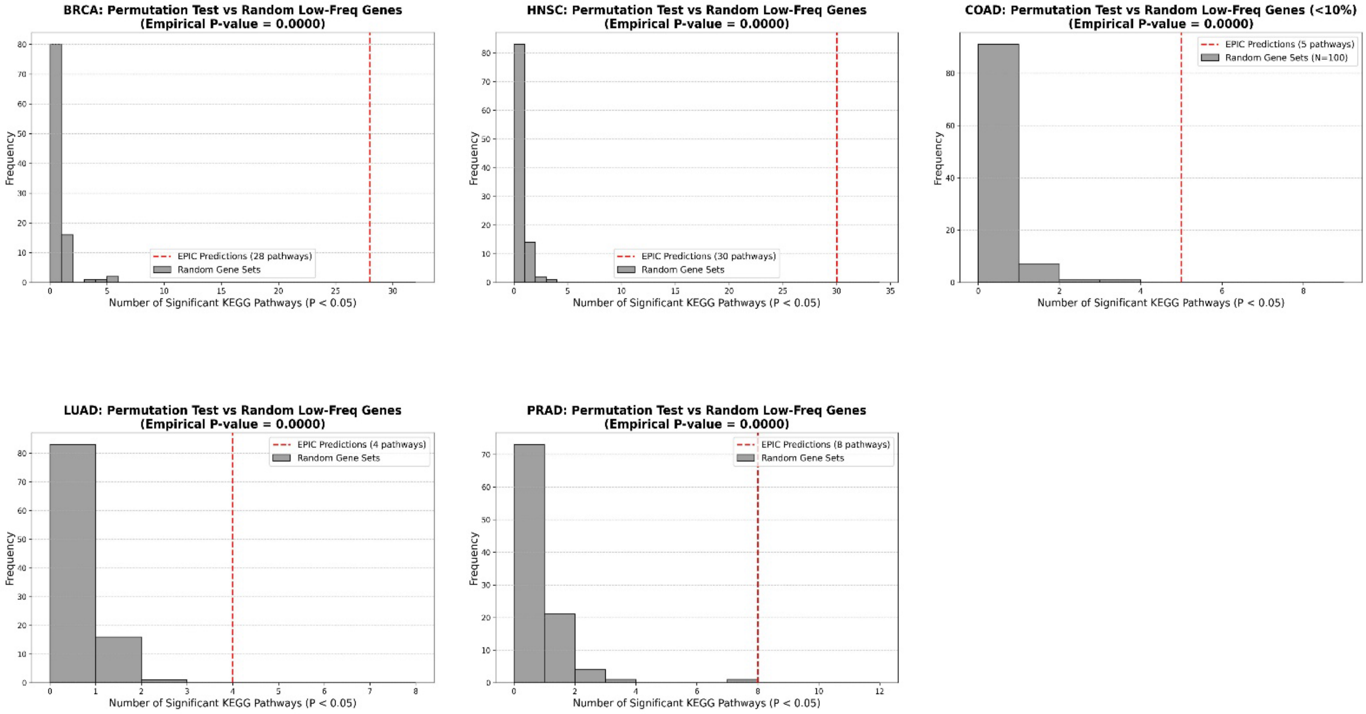

Figure S5. Statistical validation of functional enrichment via permutation tests. The gray histograms represent the null distribution of the number of significant KEGG pathways (P < 0.05) obtained from 100 random gene sets matched by mutation frequency (<5% for BRCA, HNSC, LUAD, PRAD; <10% for COAD). The red dashed lines indicate the pathways enriched by EPIC-prioritized rare driver candidates. Across all cohorts, EPIC's predictions yielded significantly stronger functional signals compared to the random null models (empirical P = 0.0), demonstrating that the model effectively isolates biologically relevant drivers from stochastic background noise.

As illustrated in Figure S5, the EPIC-prioritized rare driver candidates exhibited significantly higher functional coherence compared to the random null models across all five cancer types. The random gene sets (gray histograms) consistently failed to form significant functional clusters, typically yielding between 0 and 2 significant KEGG pathways. This confirms that low-frequency variants in cancer genomes are predominantly composed of stochastic passenger mutations with minimal biological convergence. In contrast, the candidates prioritized by EPIC (red dashed lines) consistently mapped to a profoundly larger number of pathways (e.g., 28 in BRCA, 30 in HNSC). Even in COAD and LUAD, where the global signal was more dispersed, EPIC's results remained far outside the random distribution. Across all 100 permutations for every cohort, not a single random trial reached the enrichment strength of EPIC's predictions, resulting in an empirical P-value of < 0.01 (0.0 based on 100 iterations).

These results demonstrate that the functional signals identified in EPIC's rare driver predictions are not artifacts of background mutation frequencies or stochastic noise. Instead, they represent a statistically significant selection of genes that collectively contribute to oncogenic processes. By successfully isolating signal from noise, EPIC provides a robust framework for discovering individualized driver mechanisms that are biologically plausible and statistically distinct from random mutational events.

Supplementary 9. Biological Plausibility of Predicted Novel Driver Candidates

While NCG 6.0 provides a high-confidence consensus of cancer drivers at the population level, it may not encompass all patient-specific or context-dependent functional variants. We analyzed the top-ranked genes predicted by EPIC that were not listed in the NCG 6.0 ground truth to assess their potential oncogenic roles. By cross-referencing these candidates with literature and functional databases, we observed that many of these "novel" genes demonstrate significant pathway coherence and reported associations with cancer progression.

Table S5. Literature evidence for representative novel driver candidates prioritized by EPIC.

Cancer	Pathway	Novel Candidate	Reported Biological Role & Evidence	Reference
$BRCA$	Endocrine resistance	MTOR	Acts as a central node in bypass signaling pathways that drive resistance to endocrine therapy.	[1]
COAD	TGF-beta signaling	RGMA	Functions as a BMP/TGF-β co-receptor; its genetic or epigenetic inactivation is reported to be associated with enhanced colon cancer cell proliferation and invasion.	[2]
$HNSC$	PI3K-Akt signaling	MET	Implicated in HNSC progression and as a mechanism of resistance to EGFR inhibitors.	[3]
$LUAD$	Adherens junction	FYN	Inhibits EMT and cell invasion via PI3K/AKT pathway down-regulation; high expression correlates with favorable prognosis in LUAD.	[4]
$PRAD$	Focal adhesion	VCL	Linked to increased cell migration and invasive phenotypes; serves as a potential metastatic biomarker.	[5]

References for Supplementary 9

[1] Paplomata, Elisavet, and Ruth O'Regan. "The PI3K/AKT/mTOR pathway in breast cancer: targets, trials and biomarkers." *Therapeutic advances in medical oncology* 6.4 (2014): 154-166.

[2] Li, Vivian SW, et al. "Frequent inactivation of axon guidance molecule RGMA in human colon cancer through genetic and epigenetic mechanisms." *Gastroenterology* 137.1 (2009): 176-187.

[3] Hartmann, Stefan, Neil E. Bhola, and Jennifer R. Grandis. "HGF/Met signaling in head and neck cancer: impact on the tumor microenvironment." *Clinical Cancer Research* 22.16 (2016): 4005-4013.

[4] Xue, Feng, Yong Jia, and Jian Zhao. "Overexpression of FYN suppresses the epithelial-to-mesenchymal transition through down-regulating PI3K/AKT pathway in lung adenocarcinoma." *Surgical Oncology* 33 (2020): 108-117.

[5] Ai, Jianzhong, et al. "Vinculin and filamin-C are two potential prognostic biomarkers and therapeutic targets for prostate cancer cell migration." *Oncotarget* 8.47 (2017): 82430.
